# Apurinic/Apyrimidinic Endonuclease 1 and Tyrosyl-DNA Phosphodiesterase 1 Prevent Suicidal Covalent DNA-Protein Crosslink at Apurinic/Apyrimidinic Site

**DOI:** 10.3389/fcell.2020.617301

**Published:** 2021-01-11

**Authors:** Natalia A. Lebedeva, Nadejda I. Rechkunova, Anton V. Endutkin, Olga I. Lavrik

**Affiliations:** ^1^Institute of Chemical Biology and Fundamental Medicine, Novosibirsk, Russia; ^2^Department of Natural Sciences, Novosibirsk State University, Novosibirsk, Russia

**Keywords:** apurinic/apyrimidinic site, 8-oxoguanine-DNA glycosylase, AP endonuclease 1, tyrosyl-DNA phosphodiesterase 1, poly(ADP-ribose) polymerases, DNA-protein crosslinks

## Abstract

Bifunctional 8-oxoguanine-DNA glycosylase (OGG1), a crucial DNA-repair enzyme, removes from DNA 8-oxo-7,8-dihydroguanine (8-oxoG) with following cleavage of the arising apurinic/apyrimidinic (AP) site. The major enzyme in eukaryotic cells that catalyzes the cleavage of AP sites is AP endonuclease 1 (APE1). Alternatively, AP sites can be cleaved by tyrosyl-DNA phosphodiesterase 1 (TDP1) to initiate APE1-independent repair, thus expanding the ability of the base excision repair (BER) process. Poly(ADP-ribose) polymerase 1 (PARP1) is a regulatory protein of DNA repair. PARP2 is also activated in response to DNA damage and can be regarded as the BER participant. Here we analyze PARP1 and PARP2 interactions with DNA intermediates of the initial stages of the BER process (8-oxoG and AP-site containing DNA) and their interplay with the proteins recognizing and processing these DNA structures focusing on OGG1. OGG1 as well as PARP1 and PARP2 form covalent complex with AP site-containing DNA without borohydride reduction. AP site incision by APE1 or TDP1 removal of protein adducts but not proteins’ PARylation prevent DNA-protein crosslinks.

## Introduction

Cellular DNA is continuously exposed to both endogenous and exogenous damaging agents. Oxidative stress arising from endogenous reactive oxygen species and some environmental factors, e.g., UV irradiation, is a major source of DNA damage. As a consequence, modified bases, apurinic/apyrimidinic (abasic or AP) sites, and single-strand breaks (SSBs) are generated (Cadet et al., 1999; Lesher et al., 2002). One of the major base lesions induced by oxidative stress is 8-oxoguanine (8-oxoG), which is recognized and excised by a specific DNA glycosylase, initiating the base excision repair (BER) pathway (Radicella et al., 1997). Human 8-oxoguanine-DNA glycosylase (OGG1) plays a major role in the BER pathway by removing 8-oxoguanine base lesions from oxidized G/C base pairs generated an abasic site as the major product, that is subsequently incised by AP-endonuclease 1 (APE1) and further processed by subsequent enzymes in the BER pathway (Brooks et al., 2013). Alternatively, AP site can be cleaved by tyrosyl-DNA-phosphodiesterase 1 (TDP1) to initiate APE1-independent repair, thus expanding the ability of the BER process (Lebedeva et al., 2011, 2013). In addition to the major glycosylase activity, OGG1 also possesses a minor AP lyase activity arising from the formation of a Schiff base linkage between a conserved active site lysine and the C1′ of the damaged site (Girard et al., 1997). The lyase activity eliminates the 3′ phosphate of the AP site and yields a single strand break, but this activity is thought be minor under physiological conditions (Vidal et al., 2001; Kuznetsov et al., 2005; Morland et al., 2005; Dalhus et al., 2011; Faucher et al., 2012).

AP-endonuclease 1 stimulates turnover of several glycosylases by accelerating rate-limiting product release (Esadze et al., 2017). A stimulation mechanism involving stable protein-protein interactions between free APE1 and OGG1, or the DNA bound forms, was excluded using protein crosslinking assays. APE1 capability to access the AP site without forming specific interactions with the glycosylase provides a simple and elegant mechanism to passing along unstable intermediates in BER (Esadze et al., 2017). AP-endonuclease activity of APE1 is essential for stimulation and direct interactions between APE1 and OGG1 facilitate displacement of OGG1 (Sidorenko et al., 2007, 2008).

Poly(ADP-ribose) polymerase 1 (PARP1) is a regulatory protein involved in many different processes of DNA and RNA metabolism, including DNA repair, especially those containing AP sites and strand breaks that are common intermediates in the BER pathway (Schreiber et al., 2006; Hassa and Hottiger, 2008). Upon binding to these lesions, PARP1 becomes activated for synthesis of poly(ADP-ribose) (PAR), and PARP1 can PARylate itself as well as other proteins involved in DNA metabolism (D’Amours et al., 1999). Previously, PARP1 was found capable of forming a covalent DNA-protein crosslink (DPC) at the AP site in double-stranded DNA (Khodyreva et al., 2010). The C1′ atom of the AP site participates in Schiff base formation with a lysine side chain in PARP1, and a covalent bond is formed upon reduction of the Schiff base.

8-oxoguanine-DNA glycosylase interacts with PARP1 (Hooten et al., 2011). Authors found that OGG1 binds directly to PARP1 through the N-terminal region of OGG1, and this interaction is enhanced by oxidative stress. Furthermore, OGG1 binds to PARP1 through its BRCA1 C-terminal (BRCT) domain. OGG1 stimulates the poly(ADP-ribosyl)ation activity of PARP1, which may explain the lack of poly(ADP-ribosyl)ation in cells with decreased level of OGG1. Alternatively, OGG1 expression may enhance only PARP1 automodification rather than influencing PAR formation on target proteins. Importantly, activated PARP1 inhibits OGG1. OGG1 binding to PARP1 plays a functional role in the repair of oxidative DNA damage.

PARP2 is also activated in response to DNA damage and can be regarded as the BER participant. Biochemical studies revealed that PARP2, like PARP1 (Moor et al., 2015), interacts with the BER repair factors XRCC1, DNA polymerase β and DNA ligase III (Amé et al., 1999; Schreiber et al., 2002). However, PARP2 role in the BER process is still under investigation. PARP1 and PARP2 can heterodimerize, but they recognize different targets within BER DNA intermediates (Schreiber et al., 2002; Sukhanova et al., 2019). PARP2 preferentially binds gaps or flap structures that allow suggesting PARP2 is probably involved in the later steps of the repair process (Mortusewicz et al., 2007).

Here we analyze PARP1 and PARP2 interactions with DNA intermediates of the initial stages of the BER process (8-oxoG and AP-site containing DNA) and their interplay with the proteins recognizing and processing these DNA structures focusing on OGG1. OGG1 as well as PARP1 and PARP2 form covalent complex with AP site-containing DNA without borohydride reduction. AP site incision by APE1 or TDP1 removal of protein adducts but not proteins’ PARylation prevent DPC without borohydride trapping.

## Methods

### Protein Crosslinking to AP Site

The reaction mixtures (10 μl) contained 10 nM 5′-[^32^P]-labeled DNA substrate, 50 mM Tris–HCl (pH 7.5), 50 mM Na1Cl, 1 mM DTT, 50 nM OGG1 and different concentrations of APE1 or PARP1/2. DNA duplex containing dUMP was preliminary incubated with UDG (0.5 U/μl) for 15 min at 37°C to generate AP site. Reactions were carried out at 37°C for 30 min and stopped with Laemmli loading buffer containing 5% SDS, 5% 2-mercaptoethanol, 0.3 M Tris–HCl, pH 7.8, 50% glycerol and 0.015% bromophenol blue and heated for 5 min at 95°C. The reaction products were separated by Laemmli electrophoresis in a 10% SDS-PAG followed by autoradiography and visualized by phosphorimaging using a Biomolecular Imager Typhoon FLA 9500 (GE Healthcare).

The sequences of the oligonucleotides used in experiments were as follows:

**Table:** 

AP-DNA	5′-^∗^GGCGATTAAGTTGGG**U**AACGTCAGGGTCTTCC-3′ 3′- CCGCTAATTCAACCCGTTGCAGTCCCAGAAGG-5′
8-oxoG-DNA	5′-^∗^CTCTCCCTTC**X**CTCCTTTCCTCT-3′ 3′-GAGAGGGAAGCGAGGAAAGGAGA-5′ ^∗^ – ^32^P X = 8-oxoG

### Protein Poly(ADP-ribosyl)ation

Poly(ADP-ribosyl)ation of the proteins was performed as described in Maltseva et al. (2018). The reaction mixtures (10 μl) containing 50 mM Tris–HCl (pH 7.5), 50 mM NaCl, 1 mM DTT, 2.5 μM [^32^P]-NAD^+^, 10 nM DNA, 100 nM PARP1/2 and the indicated concentrations of OGG1 and APE1 were incubated at 25°C for 20 min. Samples were supplemented with Laemmli loading buffer and heated. The reaction products were separated by Laemmli electrophoresis in a 10% SDS-PAG followed by autoradiography and visualized by phosphorimaging.

### EMSA Analysis of DNA-Protein Complexes

Protein binding to 5′-[^32^P]-DNA were analyzed in a mixture (10 μl) containing 50 mM Tris–HCl, pH 7.5, 50 mM NaCl, 10 nM 5′-[^32^P]-DNA and indicated concentrations of PARP1/2 or OGG1. The reaction mixtures were incubated at 25°C for 20 min. Loading buffer (1/5 volume) containing 20% glycerol and 0.015% bromophenol blue was then added to the samples. The DNA-protein complexes were analyzed by electrophoresis in a 5% polyacrylamide gel (acrylamide/bis-acrylamide = 60:1) in TBE buffer at 4°C followed by phosphorimaging.

## Results

### Influence of APE1 and PARP1/2 on the OGG1 Crosslinking to AP Site

8-oxoguanine-DNA glycosylase as well as PARP1 and PARP2 interact with the AP site forming Schiff base which reduction by NaBH_4_ results in covalent adducts (Nazarkina et al., 2007; Khodyreva et al., 2010; Kutuzov et al., 2015). Besides, PARP1 was shown to form covalent complex with AP site-containing DNA without NaBH_4_ reduction (Prasad et al., 2014). Here we examined whether formation of stable DNA-protein crosslink (DPC) in the absence of NaBH_4_ is the unique feature of PARP1 or other AP site-binding proteins are also able to form such adducts.

**FIGURE 1:**
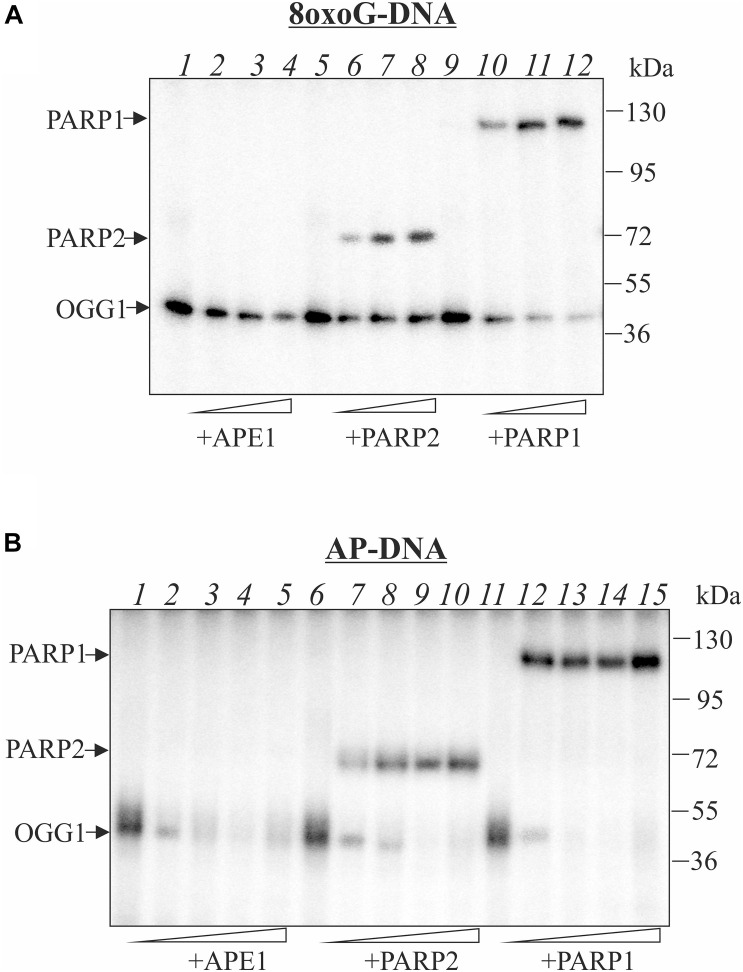
Influence of APE1, PARP2, and PARP1 on the crosslinking of OGG1 to AP site-containing DNA. Phosphorimages of crosslinked proteins to ^32^P-labeled 8-oxoG-containing DNA **(A)** and to AP site-containing DNA after treatment with UDG **(B)**. Reaction mixture included 10 nM DNA, 50 nM OGG1 and different concentrations of APE1 or PARP1/2 (50, 100, and 200 nM in **A**) and (10, 50, 100, and 200 nM in **B**). APE1, PARP1/2 were added to the reaction mixture simultaneously with OGG1.

8-oxoguanine-DNA glycosylase was incubated with the 5′-labeled DNA substrates containing an 8-oxoG or AP site generated after uracil removing by the UDG and the complex was analyzed without NaBH_4_ reduction (Figure 1). Incubation of OGG1 with DNA substrate containing 8-oxoG lesion (8oxoG-DNA) revealed the formation of DNA-protein covalent adducts in the absence of NaBH_4_ reflecting the formation of the intermediate of the DNA glycosylase/AP lyase reaction (Figure 1A). The addition of APE1 at concentrations 2 and 4 times higher than OGG1 decreased the amount of the crosslinking products (Figures 1A, lanes 2–4) which indicates a possible competition between proteins for the AP site arising from 8-oxoG excising by OGG1. The simultaneous addition of OGG1 and APE1, even at concentrations lower than OGG1, to the reaction mixture with DNA containing the AP site generated by UDG (AP-DNA) almost completely inhibited OGG1 crosslinking to DNA (Figure 1B, lanes 2–4). Under the same conditions, no APE1 crosslinks to DNA substrates were observed, which was expected because the enzyme has extremely high AP-endonuclease activity that led to almost instantaneous processing of the AP-DNA (Supplementary Figure S1).

Addition of PARP1 or PARP2 at the increased concentrations affects OGG1 crosslinking to the AP site. This effect was more pronounce in the case of AP-DNA than with the 8oxoG-DNA: both PARPs completely inhibited OGG1-AP-DNA crosslinking at 100 nM concentration, whereas in the case of 8oxoG-DNA OGG1-DNA adducts were observed even in the presence of 200 nM PARPs (compare corresponding lanes on Figures 1A,B). Although PARPs themselves effectively crosslink to the AP site generated both by OGG1 (Figure 1A) and by UDG (Figure 1B), they compete with OGG1 more efficiently on DNA with the pre-formed AP site that allows suggesting PARPs facilitate OGG1 removal from its product. Neither OGG1 nor PARPs form crosslinks with intact DNA duplex (Supplementary Figure S2).

### Effect of the TDP1 and APE1 on the OGG1 Crosslinking to AP Site

**FIGURE 2:**
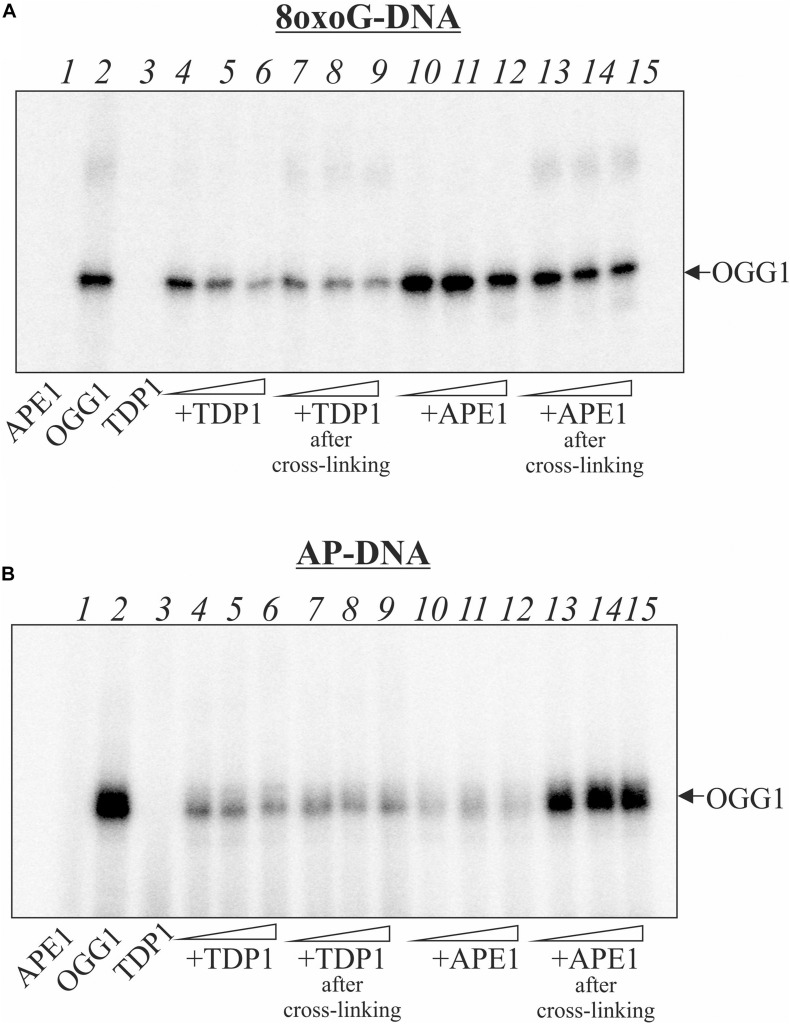
Effect of the order of TDP1 and APE1 addition on the crosslinking of OGG1 to AP site-containing DNA. Phosphorimages of crosslinked proteins to ^32^P-labeled 8-oxoG-containing DNA **(A)** and to AP site-containing DNA after treatment with UDG **(B)**. Reaction mixture included 10 nM DNA, 50 nM OGG1 and different concentrations (50, 100, and 200 nM) of TDP1 or APE1. TDP1 or APE1 were added to the reaction mixture simultaneously with OGG1 (lanes 4–6 and 10–12) or after incubation of DNA with OGG1 for 10 min (lanes 7–9 and 13–15). Lane 1 in **(A,B)** represent DNA substrate incubated with 200 nM APE1, lane 2 – DNA substrate incubated with 50 nM OGG1, lane 3 – DNA substrate incubated with 200 nM TDP1.

Earlier, we have shown that AP site is processed by tyrosyl-DNA phosphodiesterase 1 (Lebedeva et al., 2011) which can initiate APE1-independent BER pathway (Lebedeva et al., 2013). Here we performed comparative analysis of the TDP1 and APE1 effects on OGG1 crosslinking to AP site (Figure 2). Like APE1, TDP1 does not crosslink to AP site but competes with OGG1 for the AP site, thereby reducing the amount of the OGG1-DNA suicidal complexes especially on AP-DNA (Figure 2 compare lanes 4–6 on A and B panels with lane 2). The addition of TDP1 to the reaction mixture after the formation of the OGG1-DNA adducts also led to a decrease in the observed OGG1 crosslinking products with both 8oxoG- and AP-DNA (lanes 7–9). We assume that TDP1 not only competes for DNA but also is able to remove OGG1 from the DNA-protein adduct. APE1 has less than TDP1 effect on 8oxoG-DNA both in the case of simultaneous addition and when it was added after OGG1-DNA crosslink formation (Figure 2A, compare lanes 10–12 with 4–6 and lanes 13–15 with 7–9). More visible decrease in the level of the OGG1-DNA products in the case of APE1 addition to pre-formed crosslinks (lanes 13–15) may be explained by the partial hydrolysis of AP sites generated by OGG1 before APE1 addition. In the case of AP-DNA, APE1 addition to the reaction mixture after the formation of the OGG1-DNA covalent complex has little effect on the product amount (Figure 2B, lanes 13–15) in comparison with simultaneous presence of both proteins (lanes 10–12) when crosslinking is dramatically reduced. This result reflects effective hydrolysis of AP site by APE1.

### Effect of PARylation Catalyzed by PARP1/2 on the Protein Crosslinking to AP Site

**FIGURE 3:**
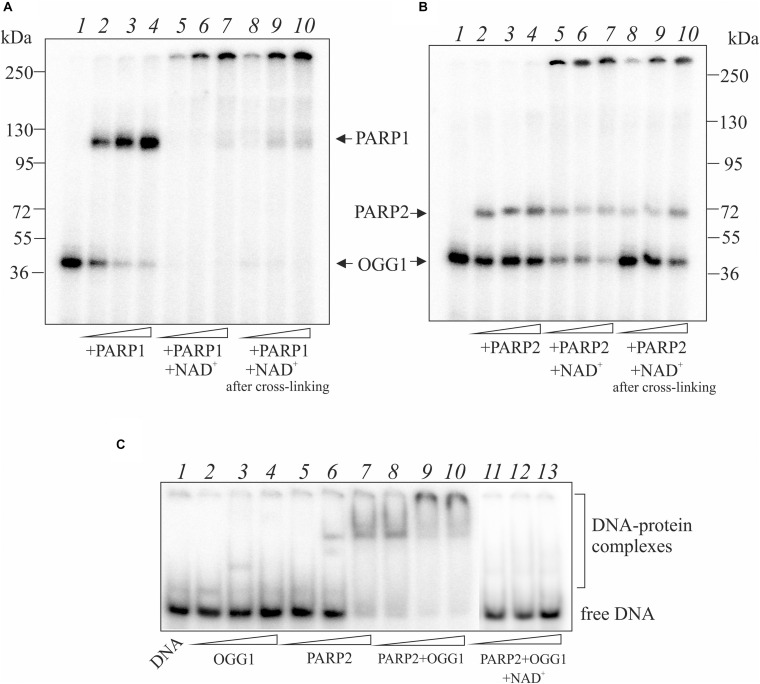
Effect of NAD^+^ addition on the crosslinking of OGG1 to AP site-containing DNA in the presence of PARP1 **(A)** and PARP2 **(B)**. Reaction mixture included 10 nM AP site-containing DNA after treatment with UDG, 50 nM OGG1 and different concentrations (50, 100, and 200 nM) of PARP1/2. NAD^+^ at final concentration 1 mM was added to the reaction mixture simultaneously (lanes 5–7) or after incubation of OGG1 with PARP1/2 for 10 min (lanes 8–10). The EMSA analysis of ternary OGG1-PARP2-DNA complex formation **(C)**. AP-DNA after treatment with UDG was titrated by OGG1 (50, 100, and 200 nM) in the absence (lanes 2–4), in the presence of 100 nM PARP2 (lanes 8–10) or 100 nM PARP2 and 1 mM NAD^+^ (lanes 11–13). Lanes 5–7 – AP-DNA titration with PARP2 (50, 100, and 200 nM) in the absence of OGG1. Lane 1 is DNA control.

The covalent attachment of OGG1 to AP site-containing DNA appears to be a suicidal event when BER is overwhelmed or disrupted. To investigate whether PARylation of OGG1 alters formation of the DPC, we performed crosslinking experiments in the presence of NAD^+^ (Figure 3). The addition of NAD^+^ to the reaction mixture with OGG1 and PARP1 led to the disappearance of OGG1 and PARP1 crosslinking to the AP-DNA (Figure 3A, lanes 5–7) and appearance of products with a low electrophoretic mobility, which suggests a possible DNA crosslinking with the PARylated OGG1 and/or PARP1. Moreover, addition of NAD^+^ to the reaction mixture after crosslinking caused very similar products (lanes 8–10), consistent with PARylation of the crosslinked PARP1/DNA complex. These experiments indicate that PARP1 auto-PARylation has no significant effect on crosslinking and conversely, crosslinking has no significant effect on PARylation catalyzed by PARP1.

When NAD^+^ was added to the reaction mixture with OGG1 and PARP2, a decrease in the amounts of crosslinked proteins was observed and products with a low electrophoretic mobility appeared (Figure 3B, lanes 5–7). The addition of NAD^+^ after preliminary crosslinking practically did not influence on the OGG1 crosslinking (Figure 3B, lanes 8–10) whereas intensity of PARP2 crosslinking products decreased approximately in the same rate as in the case of simultaneous addition of all components (compare lanes 8–10 with 5–7). Using the EMSA method (Figure 3C), complexes of OGG1 (Figure 3C, lanes 2–4, Supplementary Figure S3) and PARP2 (lanes 5–7) with DNA as well as ternary OGG1-PARP2-DNA complexes (lanes 8–10) were detected, which were destroyed by the addition of NAD^+^ (lanes 11–13). Taken together, data on PARP2/OGG1 interplay speak in favor of free but not crosslinked to DNA OGG1 is a target of PARylation catalyzed by PARP2.

**FIGURE 4:**
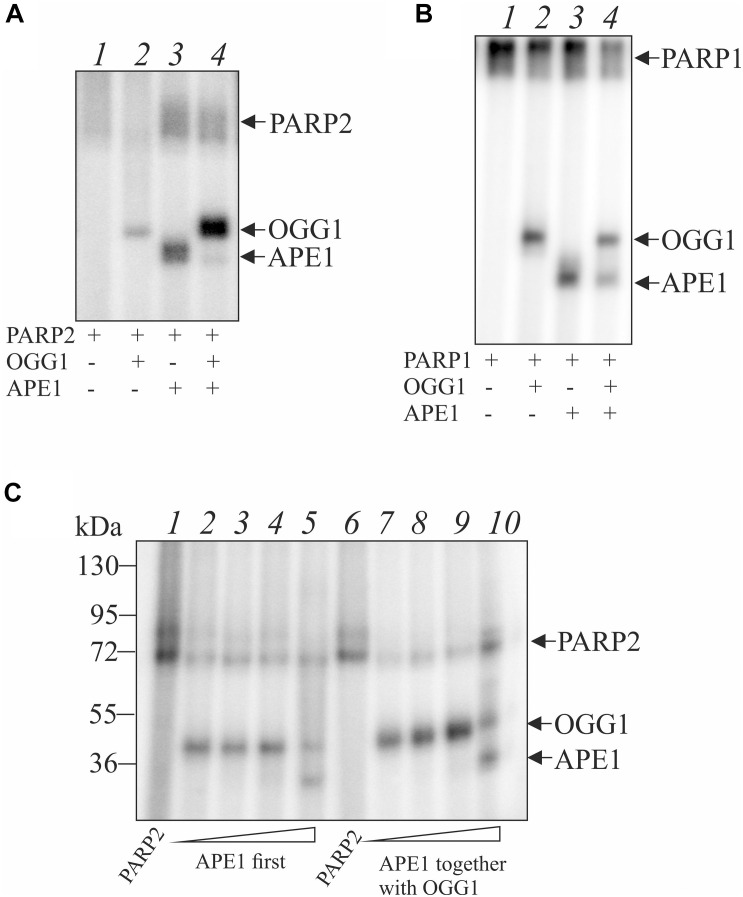
Poly(ADP-ribosyl)ation of OGG1 catalyzed by PARP1 or PARP2 in the presence of AP site-containing DNA. The reaction mixtures containing 100 nM PARP2 **(A)** or 100 nM PARP1 **(B)**, 10 nM AP-DNA after treatment with UDG and 2.5 μM [^32^P]-NAD^+^ were incubated at 25°C for 20 min in the presence 50 nM OGG1 with or without 100 nM APE1 and analyzed by SDS-PAG electrophoresis. Influence of APE1 on the OGG1 poly(ADP-ribosyl)ation by PARP2 in the presence of AP site-containing DNA **(C)**. OGG1 at a final concentration 50 nM was added to the reaction mixture after PARP2 incubation with 10, 50, 100, and 200 nM APE1 (lanes 2–5) or simultaneously with APE1 (lanes 7–10).

Indeed, OGG1 is PARylated by both PARP1 and PARP2 (Figure 4). To examine whether OGG1 may be poly(ADP-ribosyl)ated by PARP1/2, [^32^P]NAD^+^ was used as substrate for PARylation. Using DNA structure containing an AP site after UDG treatment we observed low level of OGG1 PARylation by PARP2, since PARP2 is poorly activated on double stranded DNA without break (Figure 4A, lane 2). Under the same conditions, APE1 was PARylated effectively, since it cleaved the AP site and PARP2 was activated at the break. When APE1 and OGG1 were added simultaneously, the major products corresponded to OGG1 PARylation, despite APE1 concentration was higher than OGG1 (Figure 4A, lane 4). Similar result was obtained in the case of PARylation catalyzed by PARP1 (Figure 4B). When APE1 was added first, the product of OGG1 PARylation was less intensive than in the case of the simultaneous addition of the proteins (Figure 4C, compare lanes 2–5 and 7–10). The level of OGG1 modification practically did not depend on APE1 concentration up to 100 nM: it slightly decreased with increasing APE1 concentration added first (lanes 2–4) and increased in the case of simultaneous addition of both proteins (lanes 7–9). However, at 200 nM APE1 intensity of OGG1 PARylation product decreased significantly and PARylation of APE1 was observed (lanes 5, 10).

## Discussion

Oxidative stress is one of the common sources to generate a large number of DNA damages, among DNA oxidative products 8-oxoG is the most abundant lesion, which is repaired by OGG1-initiated BER pathway. Although OGG1 is a bifunctional enzyme, its lyase activity is much lower than glycosylase that results in AP site accumulation. Recent study demonstrated that OGG1-initiated BER further increases the levels of DNA damage by generating repair intermediates, leading to PARP1 overactivation and cell death (Wang et al., 2018). In this study we show that OGG1 can generate additional DNA damages, namely DPC at AP site which form without borohydride reduction. Such DNA-protein adducts were earlier detected for PARP1 both with purified protein and in cell extracts (Prasad et al., 2014). These DNA–PARP1 crosslinks are associated with AP lyase reaction proceeding through the β-elimination (Khodyreva et al., 2010; Prasad et al., 2014). This reaction results in strand incision at C3′ and protein links to dRP group at the 3′ end. The authors have also analyzed other BER proteins including OGG1 but did not detect DPC in the absence of NaBH_4_. In our study, PARP2 DPC was also detected. It should be noted that spontaneous formation of DPC at AP site incised by OGG1 was firstly detected for XRCC1 (Nazarkina et al., 2007). Therefore, not only PARP1 but also other proteins interacting with AP site and possess AP lyase activity can form DPC without NaBH_4_ treatment.

Further, possible repair mechanism of the PARP1–DNA adducts was examined (Prasad et al., 2019). Neither APE1 no TDP1 were able to remove entire PARP1 from covalent complex with DNA. The authors proposed that proteasomal degradation of PARP1 is required for following enzymatic cleavage of covalent bond in DPC like it was earlier detected for topoisomerase 1–DNA adducts (Debethune et al., 2002). Indeed, the authors found that a proteasome inhibitor reduced repair resulting in accumulation of PARP1 DPC. Using a model DNA substrate mimicking the PARP1 DPC after proteasomal degradation, the authors demonstrated that repair was completed by the TDP1-mediated pathway of BER (Prasad et al., 2019). As OGG1 is less massive than PARP1, we proposed TDP1 capability to remove intact protein from OGG1–DNA adducts. Our results are in line with earlier observations on TDP1 implication in the release of SCAN1 mutant from the suicidal complex with DNA (Interthal et al., 2005; Kuznetsov et al., 2017). APE1 is not able to remove OGG1 from covalent complex with DNA but prevents its formation due to AP site hydrolysis (Figure 2). It was reported earlier that in the absence of NaBH_4_, PARP1 DPC was not observed for 5′-dRP-DNA generated after AP site incision by APE1 as well as APE1 was not able to process PARP1 DPC after proteasomal degradation (Prasad et al., 2014). In the case of TDP1, the cleavage products of the AP site do not contain dRP residues (Lebedeva et al., 2013); therefore they are not reactive.

Tyrosyl-DNA phosphodiesterase 1 is regarded now as the highly potential target for inhibition in cancer cells (Zakharenko et al., 2019). Commonly, TDP1 inhibitors are used in combination with Top1 poisons, thereby potentiating their effects. Our results allow speculating TDP1 inhibition as a factor to increase OGG1-mediated DNA damage in cancer cells which lost redox homeostasis resulting in oxidative DNA damage.

Poly(ADP-ribose) polymerase 1 and PARylation of repair proteins, including PARP1 itself, regulate their interaction with DNA and functional activity in the repair process. Although mutual influence of PARP1 and OGG1 was shown previously (Hooten et al., 2011), there were no data whether OGG1 is PARylated. Therefore, the present study firstly provides direct demonstration of OGG1 modification both by PARP1 and PARP2. This modification effects protein interaction with DNA but not prevent DPC formation at AP site. The model demonstrating interplay between OGG1 and APE1, TDP1, PARP1/2 on AP site-containing DNA can be hypothesized based on current observations (Supplementary Figure S4). Further investigation is required to reveal possible consequence of DPC formation in the cellular context and precise mechanism of the prevention/repair of these adducts by TDP1.

## Data Availability Statement

The original contributions presented in the study are included in the article/Supplementary Material, further inquiries can be directed to the corresponding author/s.

## Author Contributions

NL did all experiments. AE purified recombinant OGG1. NR and OL designed the study. All authors contributed to the results, discussion, and manuscript writing.

## Conflict of Interest

The authors declare that the research was conducted in the absence of any commercial or financial relationships that could be construed as a potential conflict of interest. The handling editor declared a shared affiliation and a past co-authorship with one of the authors OL at the time of review.
