# Supplementary material for: Apurinic/Apyrimidinic Endonuclease 1 and Tyrosyl-DNA Phosphodiesterase 1 Prevent Suicidal Covalent DNA-Protein Crosslink at Apurinic/Apyrimidinic Site
Source: Front Cell Dev Biol. 2021 Jan 11;8:617301. doi: 10.3389/fcell.2020.617301 (PMC7833210; doi:10.3389/fcell.2020.617301)
Supplement: Supplementary file 1 [file Table_1.DOCX]

Supplementary Material


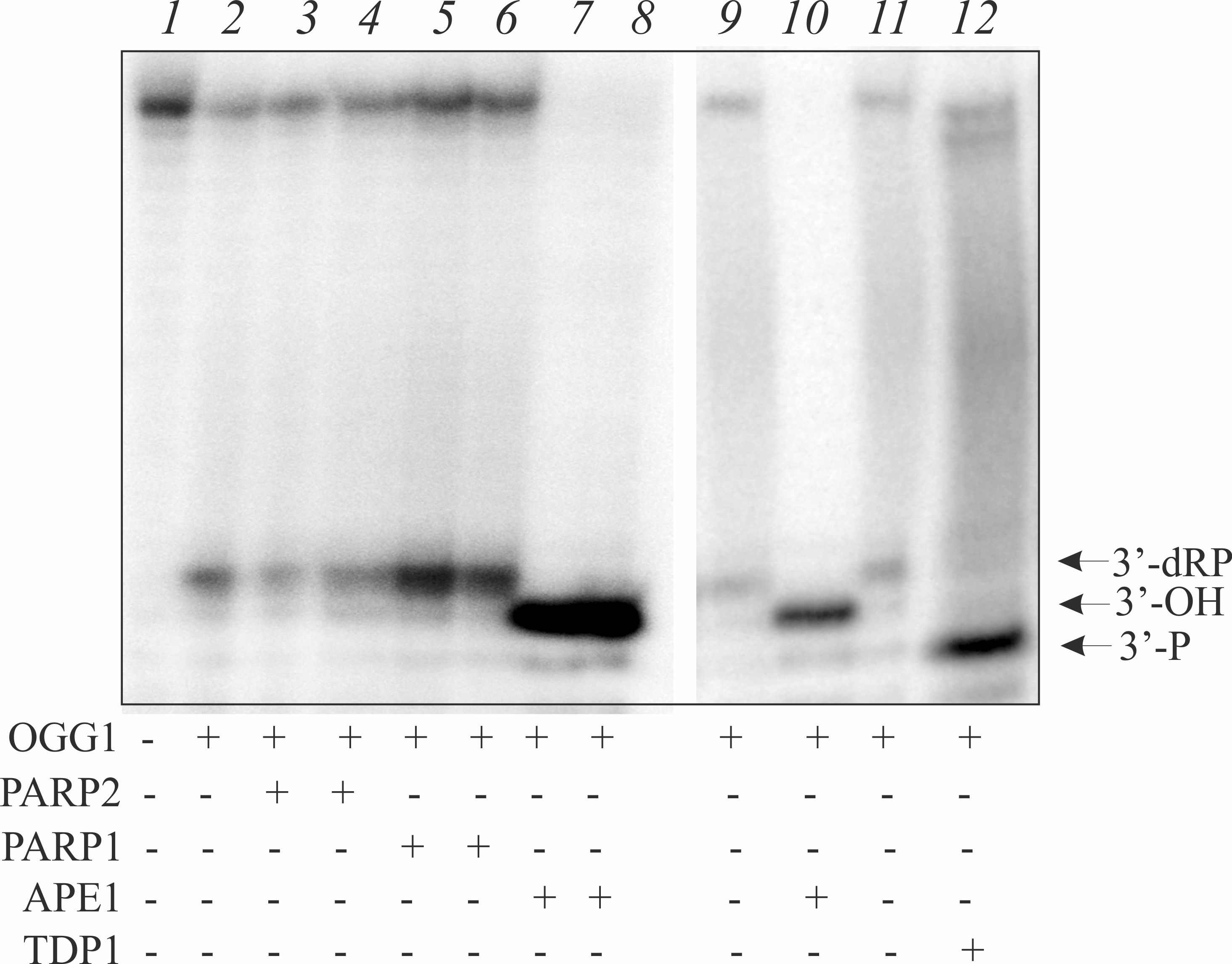


**Figure S1. Hydrolysis of AP site-containing DNA.** Reaction mixtures included 10 nM DNA, 50 nM OGG1 and different concentrations of APE1, PARP1/2 (10, 100, nM) or 100 nM Tdp1. Proteins were added after DNA incubation with OGG1 for 15 min at 37 ºC and incubated further for 15 min. Lane 1 is DNA control. The products of hydrolysis were analyzed by PAGE and followed by phosphorimaging.


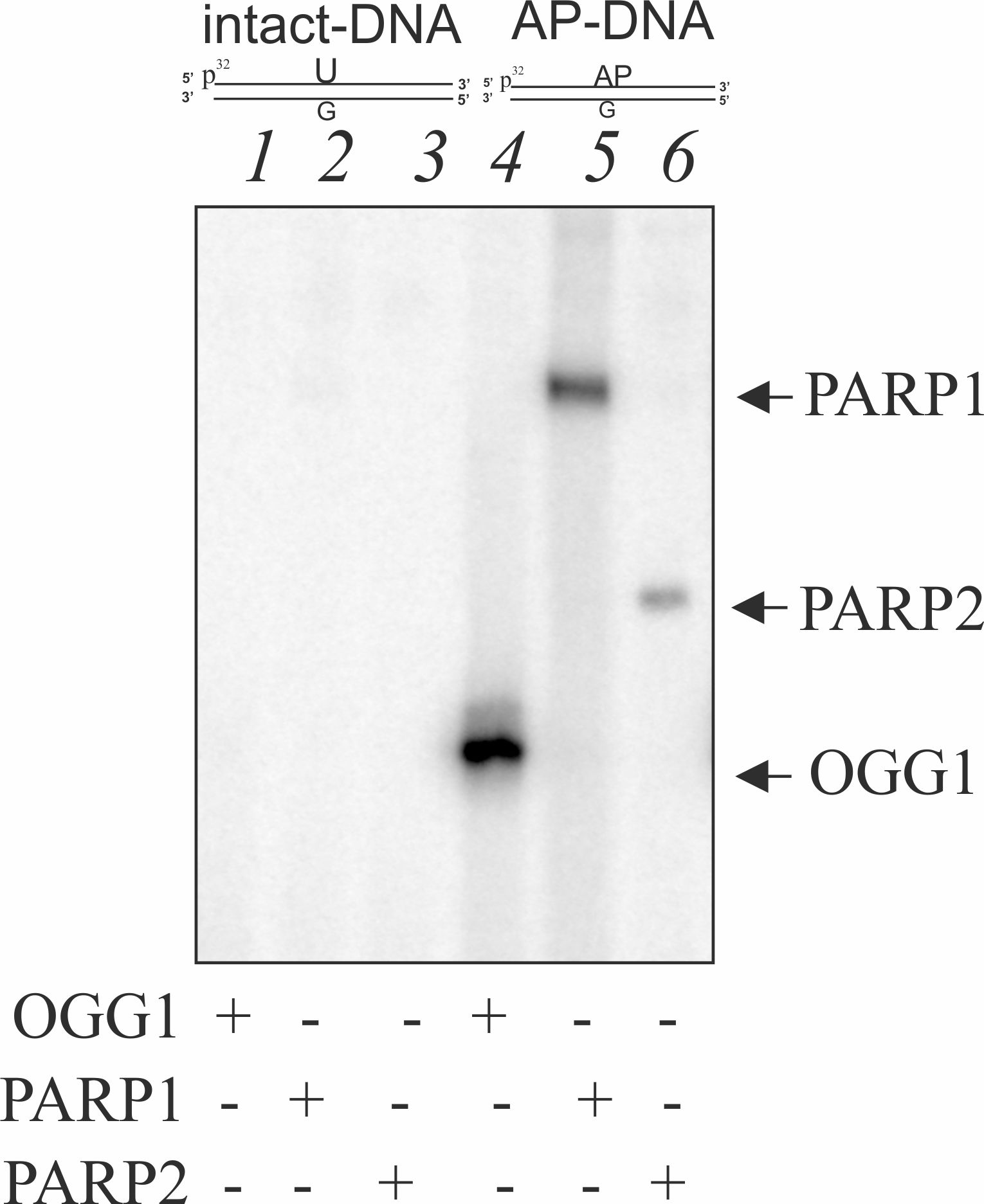


**Figure S2. OGG1 and PARP1/2 proteins cross-linked only to AP site-containing DNA.** Phosphorimages of cross-linked purified proteins to ^32^P-labeled intact-DNA (lanes 1-3) or AP site-containing DNA (lanes 4-6) after treatment with UDG are shown in picture. Reaction mixture included 10 nM DNA, 50 nM OGG1 or 50 nM PARP1/2. The cross-linking products were analyzed by SDS-PAGE and followed by phosphorimaging.


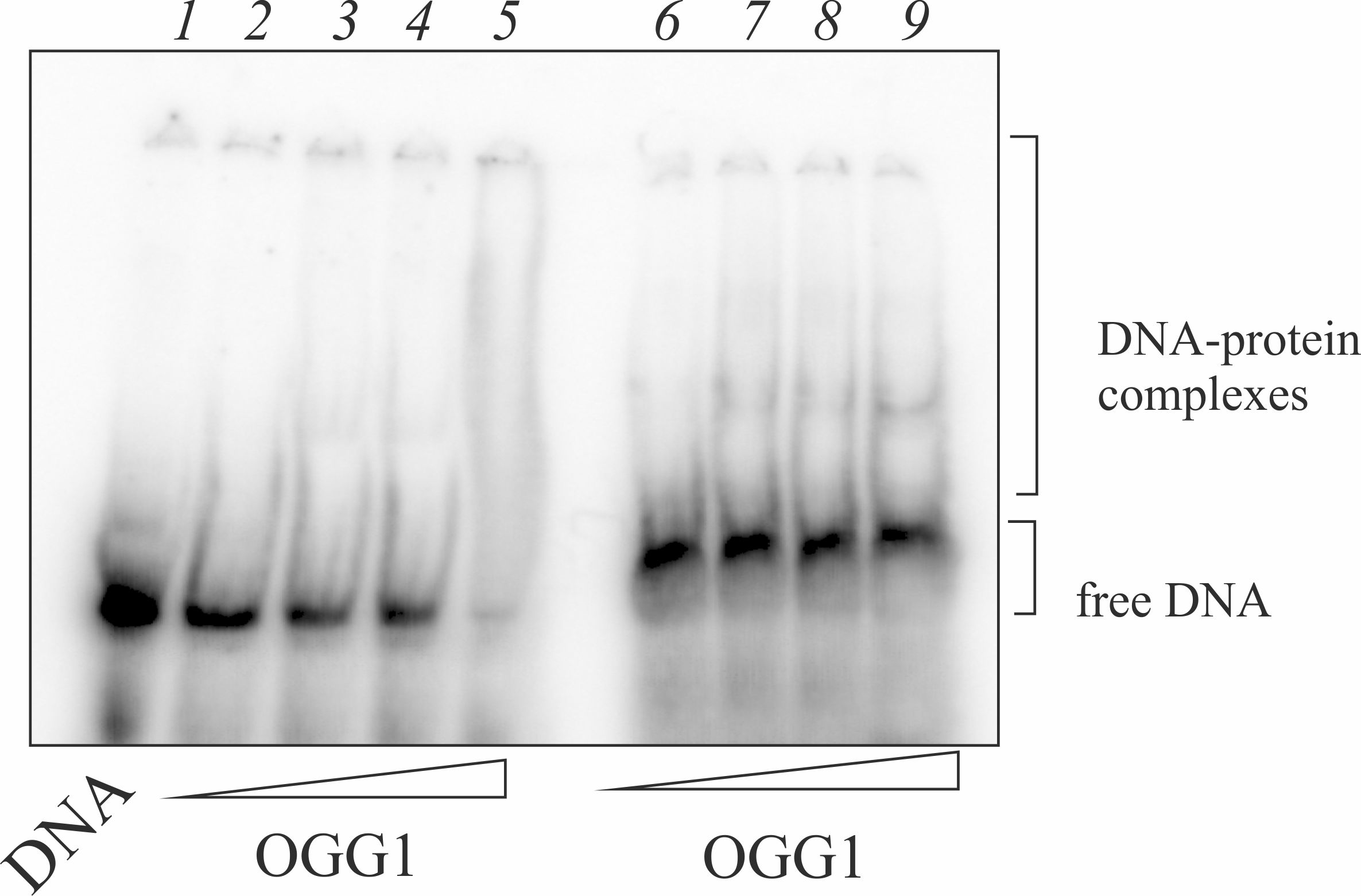


**Figure S3. The EMSA analysis of OGG1-DNA complex formation.** AP-DNA after treatment UDG was titrated by OGG1 (50, 100, 200, 500 nM). Lane 1 is DNA control. The reaction mixtures in lanes 6-9 stopped with Laemmli loading buffer containing 5% SDS, 5% 2-mercaptoethanol. The reaction mixtures containing 10 nM [^32^P]-AP-DNA and the indicated protein concentrations were incubated at 25 ºC for 20 min and analyzed by EMSA in a 5% PAG.


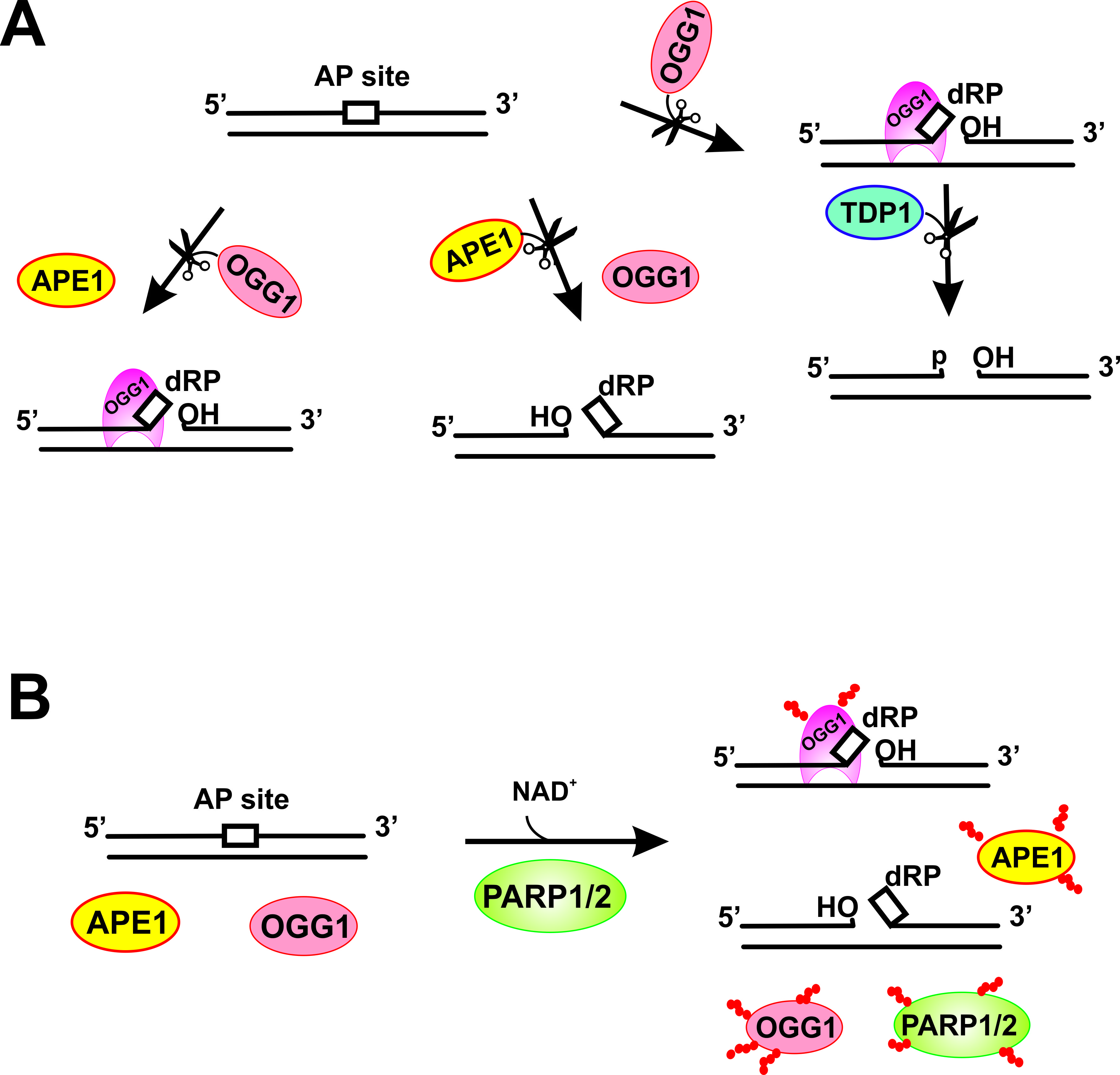


**Figure S4. Hypothetical schema of interplays between OGG1 and APE1 or TDP1 on AP-DNA (A). Proposed schema of OGG1 PARylation by PARP1/2 (B).**
